# Consensus guidelines for cellular label-free optical metabolic imaging: ensuring accuracy and reproducibility in metabolic profiling

**DOI:** 10.1117/1.JBO.30.S2.S23901

**Published:** 2025-11-11

**Authors:** Irene Georgakoudi, Melissa C. Skala, Kyle P. Quinn, Chiara Stringari, Janet E. Sorrells, Ahmed A. Heikal, Lin Z. Li, He N. Xu, Sixian You, Alex J. Walsh, Rupsa Datta, Kayvan Samimi, Amani A. Gillette, Kevin W. Eliceiri, Mihaela Balu, Stephen A. Boppart, Michelle A. Digman, Kylie R. Dunning, Conor L. Evans, Alba Alfonso Garcia, Jessica P. Houston, Wonsang Hwang, Matthew M. Lindley, Xingde Li, Zhiyi Liu, Laura Marcu, Sangeeta Murugkar, Michael G. Nichols, Raluca Niesner, Sapun H. Parekh, Narasimhan Rajaram, Suman Ranjit, Keyue Shen, Lingyan Shi, Belén Torrado, Alexander Vallmitjana, Michael Wang-Evers, Roger Zemp

**Affiliations:** a Thayer School of Engineering at Dartmouth College, Hanover, New Hampshire, United States; b Dartmouth Cancer Center, Dartmouth Health, Lebanon, New Hampshire, United States; c Tufts University, Department of Biomedical Engineering, Medford, Massachusetts, United States; d Morgridge Institute for Research, Madison, Wisconsin, United States; e University of Wisconsin, Department of Biomedical Engineering, Madison, Wisconsin, United States; f University of Arkansas, Arkansas Integrative Metabolic Research Center, Biomedical Engineering Department, Fayetteville, Arkansas, United States; g Institut Polytechnique de Paris, École Polytechnique & CNRS, Inserm, Laboratory for Optics and Biosciences, Palaiseau, France; h Washington University in St. Louis, Department of Electrical & Systems Engineering, St. Louis, Missouri, United States; i University of Illinois Urbana-Champaign, Department of Bioengineering, Urbana, Illinois, United States; j University of Minnesota Duluth, Department of Chemistry and Biochemistry, Duluth, Minnesota, United States; k University of Pennsylvania, Perelman School of Medicine, Department of Radiology, Britton Chance Laboratory of Redox Imaging, Philadelphia, Pennsylvania, United States; l Massachusetts Institute of Technology, Department of Electrical Engineering and Computer Science, Cambridge, Massachusetts, United States; m Texas A&M University, Department of Biomedical Engineering, College Station, Texas, United States; n University of Wisconsin, Department of Medical Physics, Madison, Wisconsin, United States; o University of California, Department of Dermatology and Beckman Laser Institute, Irvine, California, United States; p University of Illinois Urbana-Champaign, Beckman Institute for Advanced Science and Technology, Urbana, Illinois, United States; q University of California Irvine, Department of Biomedical Engineering, Irvine, California, United States; r The University of Adelaide, Robinson Research Institute, School of Biomedicine, Adelaide, Australia; s The University of Adelaide, Centre of Light for Life, Adelaide, Australia; t The University of Adelaide, Institute for Photonics and Advanced Sensing, Adelaide, Australia; u Massachusetts General Hospital, Wellman Center for Photomedicine, Harvard Medical School, Boston, Massachusetts, United States; v University of California, Department of Biomedical Engineering, Davis, California, United States; w New Mexico State University, Chemical & Materials Engineering, Las Cruces, New Mexico, United States; x Thayer School of Engineering at Dartmouth College, Hanover, New Hampshire, United States; y Johns Hopkins University, Biomedical Engineering Department, Baltimore, Maryland, United States; z Zhejiang University, State Key Laboratory of Extreme Photonics and Instrumentation, College of Optical Science and Engineering, International Research Center for Advanced Photonics, Hangzhou, Zhejiang, China; aa University of California, Departments of Neurological Surgery and Biomedical Engineering, Davis, California, United States; ab Carleton University, Department of Physics, Ottawa, OntarioCanada; ac Creighton University, Department of Physics, Omaha, Nebraska, United States; ad Dynamic and Functional In vivo Imaging Department of Veterinary Medicine, Freie Universitaet Berlin, Berlin, Germany & Biophysical Analytics, German Rheumatology Research Center, Berlin, Germany; ae University of Texas at Austin, Department of Biomedical Engineering, Austin, Texas, United States; af University of Arkansas, Biomedical Engineering Department, Fayetteville, Arkansas, United States; ag Arkansas Integrative Metabolic Research Center, Fayetteville, Arkansas, United States; ah Winthrop P. Rockefeller Cancer Institute, Little Rock, Arkansas, United States; ai Georgetown University, Department of Oncology, Washington, DC, United States; aj University of Southern California, Department of Biomedical Engineering, Los Angeles, California, United States; ak University of Southern California, Norris Comprehensive Cancer Center, Los Angeles, California, United States; al University of California, Shu Chien-Gene Lay Department of Bioengineering, San Diego, California, United States; am University of California Irvine, Beckman Laser Institute, Irvine, California, United States; an Massachusetts General Hospital, Cutaneous Biology Research Center, Harvard Medical School, Boston, Massachusetts, United States; ao University of Alberta, Department of Electrical and Computer Engineering, Edmonton, Alberta, Canada

**Keywords:** metabolic imaging, endogenous fluorescence, fluorescence lifetime imaging microscopy, nicotinamide adenine dinucleotide, flavin adenine dinucleotide, nicotinamide adenine dinucleotide phosphate, redox ratio, bound fraction, calibration

## Abstract

**Significance:**

Cellular metabolism plays a central role in health and disease, making its study critical for advancing diagnostics and therapies. Label-free optical metabolic imaging using endogenous fluorescence from reduced nicotinamide adenine dinucleotide (phosphate) [NAD(P)H] and flavin adenine dinucleotide (FAD) provides nondestructive, high-resolution insights into metabolic function and heterogeneity from the sub-cellular to the tissue level. Standardized approaches are essential to ensure reproducibility and comparability across studies.

**Aim:**

We aim to establish a consensus framework for the acquisition, calibration, and reporting of microscopic imaging metabolic function assessments based on fluorescence intensity and lifetime measurements of NAD(P)H and FAD.

**Approach:**

We present best practices for calibrating, analyzing, and reporting fluorescence intensity-based optical redox ratios and fluorescence lifetime data using multiexponential fitting and phasor analysis. Guidelines for validation experiments and cross-system standardization are provided to improve accuracy and reproducibility.

**Results:**

We demonstrate the importance of calibration procedures and normalization strategies for intensity-based optical redox measurements. We highlight needed calibration, signal-to-noise ratio considerations, and the impact of distinct analytical approaches on fluorescence lifetime-based metabolic function metrics.

**Conclusion:**

We recommend a consistent, practical framework for reproducible, label-free, optical metabolic imaging, facilitating robust comparisons across studies and supporting the broader adoption of optical metabolic imaging technologies for biomedical research and clinical translation.

## 1 Introduction

Investigation of cellular and tissue metabolism is pivotal for unraveling the intricate mechanisms underlying health and disease. Label-free optical imaging leveraging the intrinsic fluorescence of reduced nicotinamide The adenine dinucleotide (NADH), reduced nicotinamide adenine dinucleotide phosphate (NADPH), oxidized flavins (flavin adenine dinucleotide—FAD, and flavin mononucleotide—FMN), and oxidized flavoproteins (Fp) for imaging contrast offers unique advantages for the study of metabolism. The ability to acquire optical metabolic measurements in a nondestructive and quantitative manner presents a substantial advantage, preserving the integrity and metabolic relevance of the biological specimens. In addition, the high spatiotemporal single-cell scale resolution provided by these fluorescence-based optical imaging techniques is essential for monitoring dynamic metabolic changes and understanding the complex interactions between cells and their surrounding matrix. Furthermore, these methods circumvent the challenges posed by exogenous sources of contrast, which can introduce artifacts or possess limited sensitivity to important aspects of metabolic function.

**Fig. 1:**
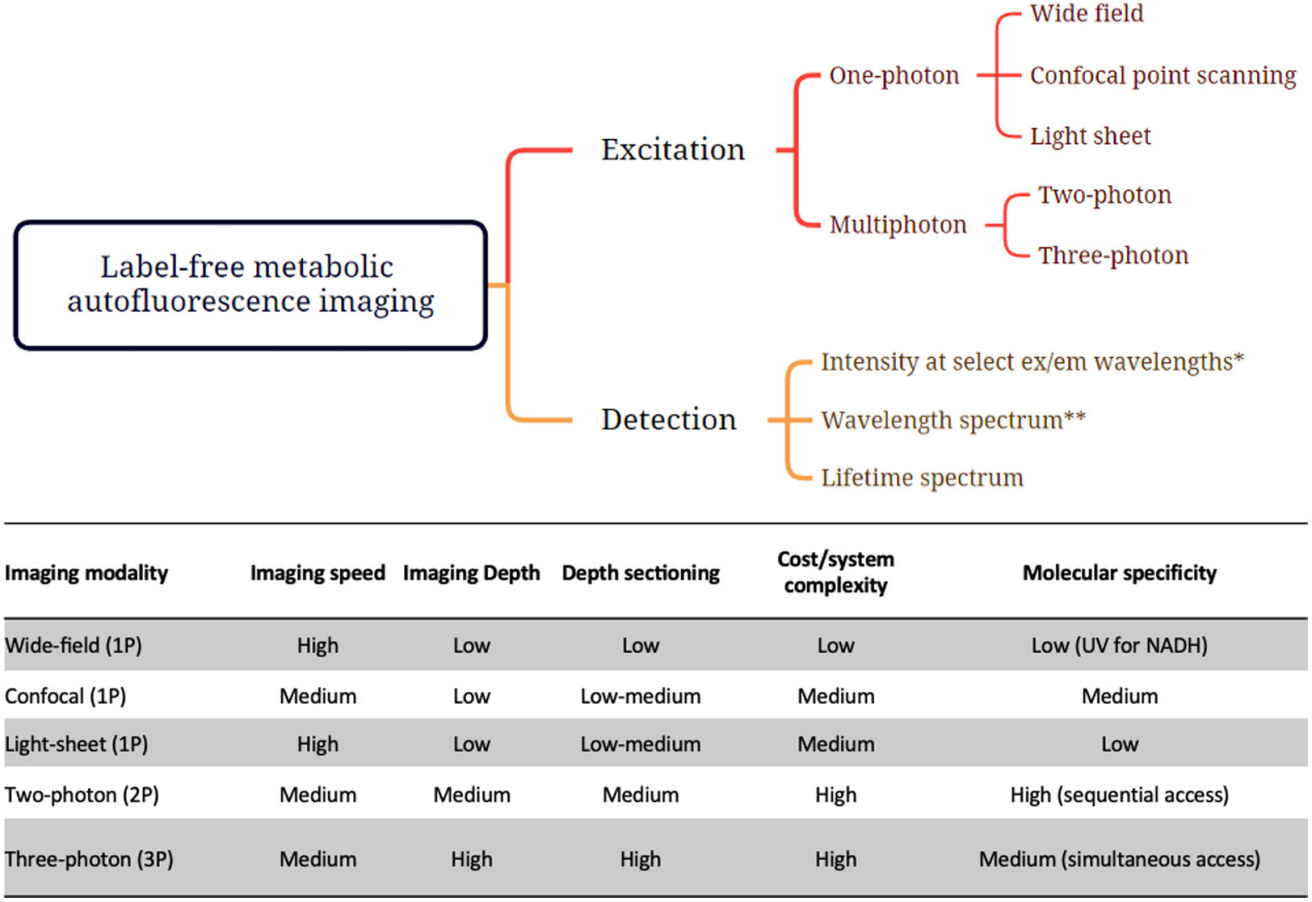
Overview of different types of endogenous fluorescence-based measurements to assess metabolic function. *Camera or point detector, collecting data at a minimum of two excitation/emission wavelength pairs for NAD(P)H and FAD measurements. **Intensity data acquired from a single point or in spectral imaging mode over a wide range of wavelengths.

A number of label-free, autofluorescence-based, optical metabolic imaging tools are being developed, including hyperspectral, polarization, and far-red fluorescence.^1^^–^^3^ Here, we focus on methods that utilize intensity and fluorescence lifetime imaging microscopy (FLIM) measurements, aiming to characterize NAD(P)H and flavin/flavoprotein emission, typically in the 400 to 550 nm range. Several published reviews cover the fundamentals and applications of these techniques.^4^^–^^10^ Different types of measurements can be performed to assess metabolic function via endogenous fluorescence, and they all have advantages and limitations; thus, the optimal optical metabolic measurement depends highly on the tissue or disease state to be evaluated (Fig. 1). As these tools gain wider adoption with the scientific community, it is important to establish a framework to understand the relevance of different optical readouts to metabolic function. This framework will also enable quantitative comparisons of results acquired over time by multiple research teams. To achieve this goal, we aim to clarify the definitions of the metrics used and their metabolic functional context. In addition, we provide recommendations for acquisition, reporting, and calibration procedures, covering both endogenous fluorescence intensity and lifetime microscopic imaging measurements.

## 2 Fluorescence Intensity-Based Measurements of Metabolic Function

### 2.1 Cellular Metabolism and Redox Potential

Label-free intensity-based fluorescence studies aiming to assess metabolic function, typically report on the cell redox state. The [NAD^+^]/[NADH] ratio, referred to as the NAD^+^ coupled redox state, was shown to be tightly controlled and thermodynamically coupled to the [ATP]/[ADP][Pi] in seminal studies performed by HA Krebs and his colleagues.^11^^–^^19^ During mitochondrial oxidative phosphorylation, the electrons flow from complex I and II at the lowest redox potential to complexes III and IV, which occupy increasingly higher redox potentials.^20^ The [NAD^+^]/[NADH] ratios inside and outside mitochondria are very different, and there are several pathways and shuttles that have evolved to maintain appropriate ratios in all compartments and ensure proper cell function.^6^ The relative flux of substrates through glycolysis, the tricarboxylic acid (TCA) cycle (also known as the Krebs cycle or citric acid cycle), fatty acid synthesis and oxidation, and amino acid catabolism (e.g., glutaminolysis) both impact and are impacted by the cytosolic and mitochondrial [NAD^+^]/[NADH] ratio.

### 2.2 Endogenous Fluorescence Intensity-Based Assessments of Cellular Metabolic State

Britton Chance and several of his colleagues established the fluorescence signatures of NADH [referred to as reduced pyridine nucleotide (PN) in their original studies] from isolated mitochondria, living cells, and tissues and investigated how they are related to intracellular metabolism, redox status, and oxygenation.^21^^–^^23^ The term NAD(P)H is used to refer to fluorescence emanating potentially from both NADH and NADPH as their excitation/emission spectra overlap. However, we note that the primary roles of the NAD^+^/NADH and NADP^+^/NADPH redox pairs are different, with the former associated with the redox potential of the cell, and the latter related to reductive biosynthesis and anti-oxidant defenses.

**Fig. 2:**
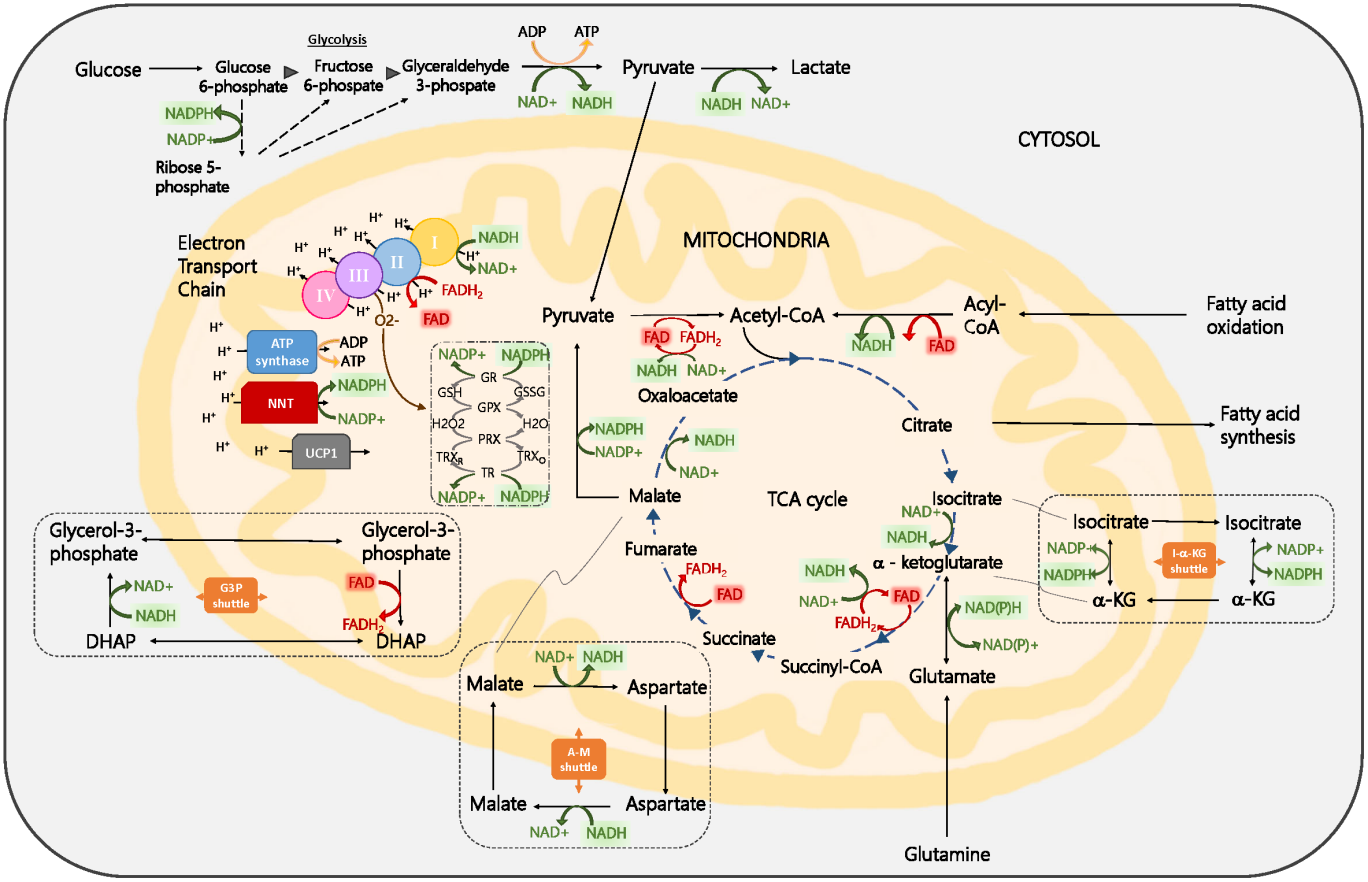
Overview of key cellular metabolic pathways in which NADH, FAD, and NADPH are involved, including glycolysis, the TCA cycle, oxidative phosphorylation, fatty acid oxidation and synthesis, and the glutathione pathway. Important shuttles such as the G3P, A-M, and I-a-KG shuttles, responsible for maintaining distinct redox states in the mitochondria and the cytosol, are also included. Abbreviations: A-M, aspartate-malate; CoA, coenzyme A; FAD, flavin adenine dinucleotide; G3P, glycerol-3-phosphate; GSH, glutathione; I-a-KG, isocitrate-a-ketoglutarate; NAD, nicotinamide adenine dinucleotide; NADH, reduced nicotinamide adenine dinucleotide; NADP, nicotinamide adenine dinucleotide phosphate; NADPH, reduced nicotinamide adenine dinucleotide phosphate; TCA, tricarboxylic acid. Reproduced with permission from Georgakoudi and Quinn.^6^

Although NAD(P)H naturally fluoresces, NAD(P)^+^ does not. Interestingly, the opposite is true for the autofluorescence emanating from flavin-associated metabolic co-enzymes (i.e., FAD, FMN, and associated flavoproteins) in cells, with oxidized moieties that yield fluorescence. Lipoamide dehydrogenase (LipDH)-associated Fp fluorescence has been found to account for more than half of the cellular flavin-associated fluorescence.^24^^–^^27^ However, free FAD as well as a number of flavoproteins containing FAD or FMN moieties may also contribute to the overall flavin autofluorescence signals. In addition to LipDH, electron transfer flavoprotein (ETF) is also another potentially significant source of Fp fluorescence. FAD or Fp are often used as the terms that refer collectively to flavin-associated fluorescence as the excitation and emission spectra of these species overlap highly and are not typically distinguished during imaging. Here, we will use the term FAD to refer to flavin-associated cellular autofluorescence. In optical redox ratio (ORR) studies, the FAD/NAD(P)H fluorescence ratio has been used as a redox equivalent of the mitochondrial NAD^+^/NADH and a more precise indicator of the mitochondrial oxido-reductive state than using NAD(P)H alone.^28^^–^^35^ FAD fluorescence is assumed to be a surrogate indicator of mitochondrial [NAD^+^]. A recent review describes in detail the reported fluorescence characteristics and metabolic functions of these fluorophores.^6^ Figure 2 outlines some of the key metabolic reactions in which NAD(P)H and FAD participate. Note that there is an overlap in the excitation and emission spectra of NAD(P)H and FAD and that incomplete separation of NAD(P)H and FAD signals can lead to erroneous redox ratio results. Careful selection of the excitation and detection of NAD(P)H and FAD may alleviate this issue.

### 2.3 Working Definitions of the Optical Redox Ratio

**Fig. 3:**
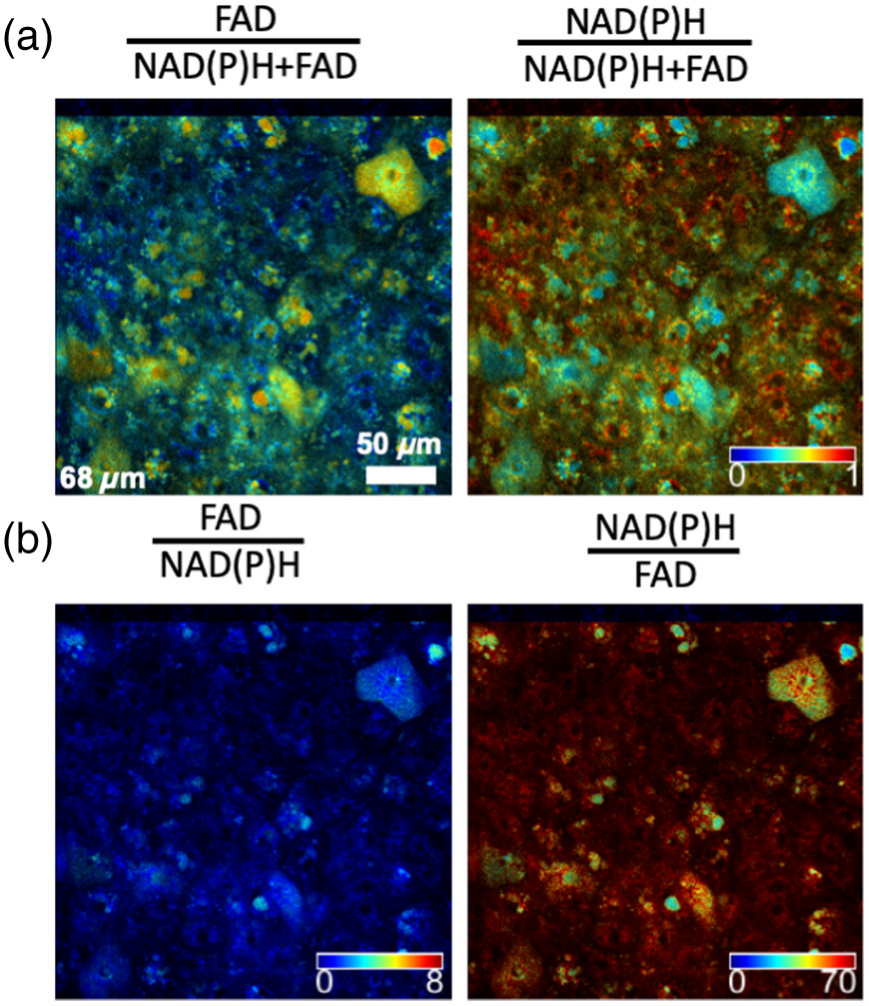
Optical redox ratio coded images from a cervical epithelial tissue acquired at a depth of 68  μm, representing the different forms of optical redox ratio reported in the literature. Redox variations within and among cells are easier to visualize in the normalized redox ratio images (top), than in the corresponding unnormalized ones (bottom). The same colorbar is used for the redox ratio-coded images of the top row. Study approved by Tufts Health Sciences Institutional Review Board (protocol #10283).

In one of the first studies to report ORR information from images as opposed to spectra, the FAD/NAD(P)H ratio was modified to its normalized form [Eq. (1)]. This normalization ensures that the redox ratio values range between 0 and 1, enabling visualization of metabolic heterogeneity^36^ (Fig. 3). The ORR has been adopted in this form by a number of groups, i.e.,

$$\text{Optical redox ratio }(\mathrm{ORR})=\frac{\mathrm{FAD}}{\mathrm{NAD}(\text{P})\text{H}+\mathrm{FAD}}.$$ (1)

This modified form usually normalizes the distribution of the redox ratio and can have advantages for image visualization and statistical analysis. The fluorescence intensity of a fluorophore is directly associated with its concentration and fluorescence quantum yield, i.e., the ratio of fluorescently emitted photons to the photons absorbed. The fluorescence quantum yield of NAD(P)H is known to increase by ∼2- to 10-fold upon protein binding, to a degree that is dependent on the binding substrate.^6^^,^^37^ Despite such variability in quantum yield, ORR measurements of this form have been shown to correlate with mass-spectrometry-based measurements of FAD/(NADH+FAD) and ${\mathrm{NAD}}^{+}/(\mathrm{NADH}+{\mathrm{NAD}}^{+})$ in epithelial tissue and mesenchymal stem cell cultures undergoing adipogenic and osteogenic differentiation.^38^^,^^39^ Measurements have also been validated with respirometry measurements using several breast cancer cell lines, lymphocytes, and the cumulus oocyte complex.^40^^–^^42^

Based on Eq. (1), an increase in the ORR indicates an increase in the ratio of oxidized to reduced molecules. This increase is typically associated with enhanced oxidative phosphorylation, glutaminolysis, and electron transport chain (ETC) uncoupling from ATP formation, or decreased TCA cycle relative to ETC activity.^43^^,^^44^ An increase in the ORR has also been shown to correlate with an increase in reactive oxygen species.^45^ Enhancements in glycolysis, fatty acid synthesis, and fatty acid oxidation have been shown to result in a decrease in the ORR (Table 1).^43^

**Table 1:** Typical changes in ORR and FLIM metrics depending on pathway activity changes (these changes can be transient and cell/tissue/environment dependent).

Metabolic pathway	$\frac{\mathrm{FAD}}{\mathrm{NAD}(\text{P})\text{H}+\mathrm{FAD}}$	$\frac{\mathrm{NAD}(\text{P})\text{H}}{\mathrm{NAD}(\text{P})\text{H}+\mathrm{FAD}}$	NAD(P)H bound fraction and/or $\tau_{m}$
↑ ETC activity	⇧	⇩	⇧
↑ glutaminolysis	⇧	⇩	⇧
↑ ETC uncoupling	⇧	⇩	⇧ or ⇩
↑ glycolysis	⇩	⇧	⇩
↑ fatty acid oxidation	⇩	⇧	⇩
↑ fatty acid synthesis	⇩	⇧	⇧
↑ TCA relative to ETC activity	⇩	⇧	⇩

Chemical investigations of NADH and the redox state report definitions of the redox ratio with either the reduced^46^^–^^49^ or oxidized^50^^–^^52^ species in the numerator. This lack of consensus on the definition of redox ratios is also present in ORR studies, where the ORR has also been reported as follows:

$$\text{Optical redox ratio }(\mathrm{ORR})=\frac{\mathrm{NAD}(\text{P})\text{H}}{\mathrm{NAD}(\text{P})\text{H}+\mathrm{FAD}}.$$ (2)

Although either Eq. (1) or Eq. (2) can be chosen to define the ORR, the directional changes are opposite. Thus, an increase in Eq. (2) indicates an increase in the ratio of the reduced to oxidized molecules. For metabolic interpretations, the ORR as defined in Eq. (2) may increase when glycolysis is enhanced, and oxidative metabolism is decreased (Table 1).^53^^–^^55^ Conversely, the ORR per Eq. (2) decreases when glycolysis is inhibited and/or oxidative metabolism is enhanced.^55^^,^^56^ Other metabolic pathways such as fatty acid synthesis and oxidation, glutaminolysis, and pentose phosphate pathway would also influence Eqs. (2) and (1). Simply put, these relationships for Eq. (2) are the opposite of Eq. (1) because the two equations can be related by [Eq. (1)] = [1 – Eq. (2)]. Thus, regardless of the equations used, the metabolic relevance of the optical outcomes has been robustly demonstrated with mass spectrometry, oxygen consumption, mitochondrial membrane potential, metabolic inhibitors, and substrate experiments.^38^^,^^39^^,^^43^^,^^55^^,^^57^^–^^59^

Independent of whether Eq. (1) or Eq. (2) is used, similar levels of contrast and heterogeneity are easily visualized in ORR images because values are constrained between 0 and 1 [Fig. 3(a)]. When using unnormalized versions of the ORR (i.e., FAD/NAD(P)H or NAD(P)H/FAD), the images may still reflect redox changes, especially when the intensities of both fluorophores are comparable; however, the range of ORR values can theoretically range from 0 to ∞, resulting in highly skewed distributions that can make metabolic heterogeneity harder to perceive and quantification or comparisons less straightforward because widely used metrics (e.g., the mean) and tests (ANOVA, t-test) assume a normal distribution [Fig. 3(b)]. Thus, the use of a normalized ORR form [Eq. (1) or Eq. (2)] is strongly preferred. Regardless of ORR definitions, it is important to note that NAD(P)H and FAD redox pairs are involved in numerous metabolic pathways (Fig. 2). Therefore, independent measurements such as extracellular flux analysis, metabolite assays, genomic, transcriptomic, proteomic, or biochemical analyses are often performed to provide complementary information to the ORR and support the interpretation of metabolic changes.

### 2.4 Calibration and Analysis Considerations

NAD(P)H and FAD imaging typically require normalization or calibration of fluorescence intensity with known fluorophores of predetermined concentrations. Factors affecting fluorescence signals such as illumination power, laser pulse width (for multiphoton excited fluorescence), transmission efficiency through filters and other optical components, detector efficiency, detector gain, and exposure or pixel dwell time can vary from study to study and in some cases among experimental groups or time points within studies. In calibrations, using standard fluorophores that are photostable, such as coumarin, rhodamine 110 or fluorescein, collected on the same day and under acquisition settings identical to NAD(P)H and FAD imaging is a reasonable approach to account for the impact of factors that can significantly affect ORR values.^40^^,^^60^^,^^61^ Alternatively, these known fluorophores can also be used to establish transfer functions capable of normalizing intensity under a range of acquisition settings used in NAD(P)H and FAD imaging, obviating the need for new calibrations for every experimental day or change in acquisition parameters.^38^^,^^39^^,^^61^^–^^63^ Reporting ORR values for a group of cells of interest relative to a corresponding control group (e.g., cells treated with a certain agent versus a vehicle control) with measurements for the two groups performed under the same settings and days is also an approach often used. However, if the values of ORR for the control group are significantly different among experiments or time points, as a result of variations in incident illumination power, for example, it may be more challenging to combine results from multiple experiments or, importantly, to interpret the origins of ORR differences between the two groups. Although these commonly used approaches can account for (or minimize) variation within an individual study, they do not correct for differences across imaging systems with different relative efficiencies of NAD(P)H and FAD fluorescence collection.

**Fig. 4:**
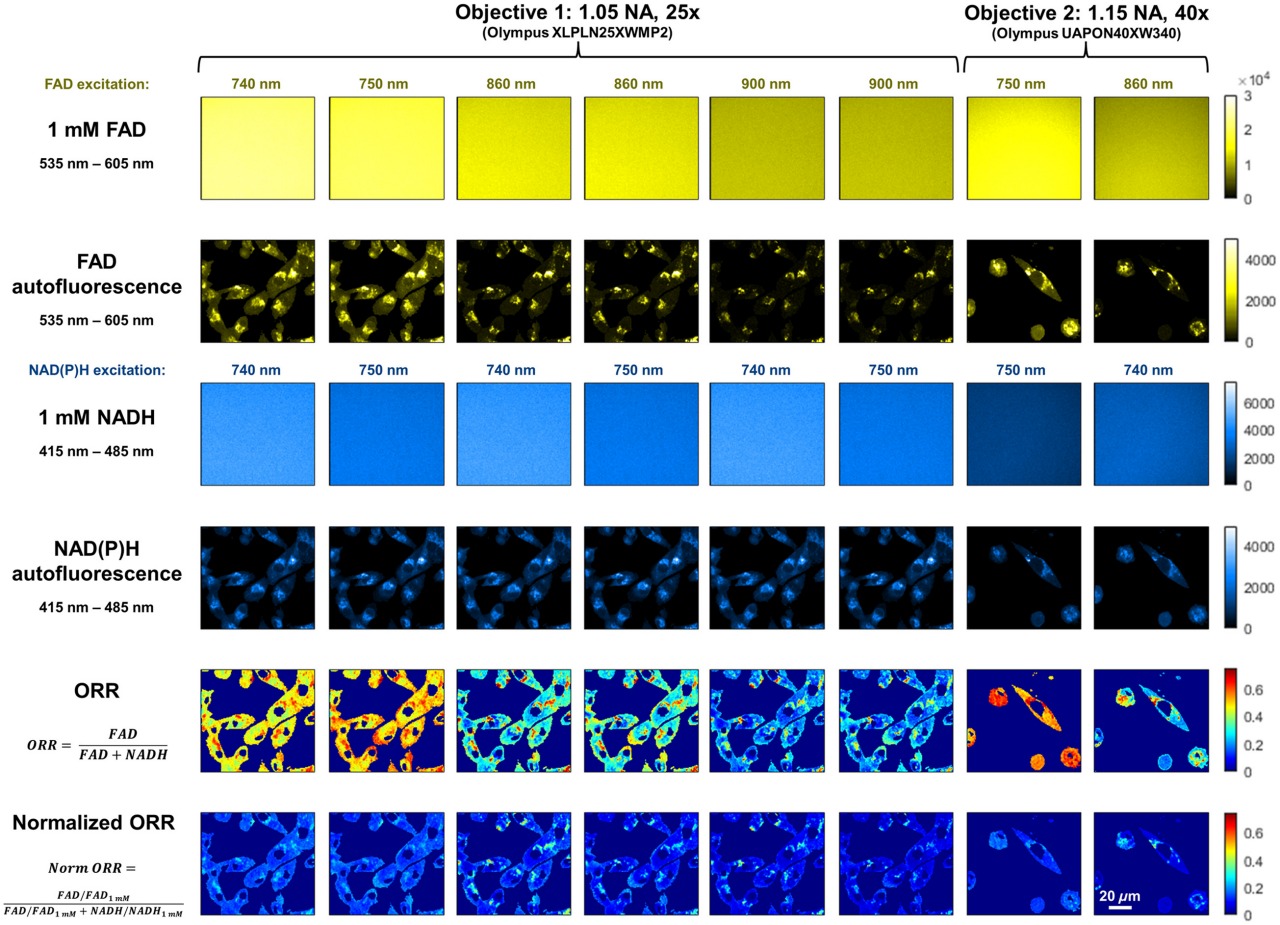
Optical redox ratio (ORR) of cells and solutions with different acquisition parameters. Human pancreatic cancer cells (MIA PaCa-2, ATCC CRL-1420) in DMEM with 10% FBS and 1% Penn/Strep antibiotic were imaged using a custom epi-detection multiphoton imaging system with 80 MHz excitation from a tunable fs laser (Insight X3+, Spectra Physics). Sequential images of FAD and NAD(P)H autofluorescence in cells were acquired using different excitation wavelengths and objective lenses, as indicated above each image. Images of 1 mM NADH and FAD solutions were additionally acquired at all acquisition and illumination conditions. ORR was calculated from FAD and NAD(P)H intensities (fifth row) or calculated with normalized intensities (sixth row) by dividing by the mean intensity of 1 mM NADH and FAD at the corresponding acquisition and illumination conditions. Images are sums of 20 frame averages at the same location, with 20 mW incident power on the sample (incident light pulse width = 170 fs; pixel dwell time=2.5  μs; frame rate = 0.19 fps, frame size=512×512  pixels/100×100  μm). Images were collected using hybrid detectors at constant gain for all measurements. Cell and nuclear areas were masked using an arbitrary threshold NAD(P)H intensity at 740 nm and that mask was used for all images from the same objective lens. Raw images from both channels were summed with a 3×3 spatial filter prior to ORR calculation, and ORR images were median filtered with a 5×5 filter to remove outliers. Note that NAD(P)H signal leaks into the FAD channel when FAD is acquired at 740 and 750 nm, which leads to a washing out of features in the ORR images. No spectral unmixing was used to account for the NAD(P)H leakage into the FAD channel but should be considered for future work.

**Fig. 5:**
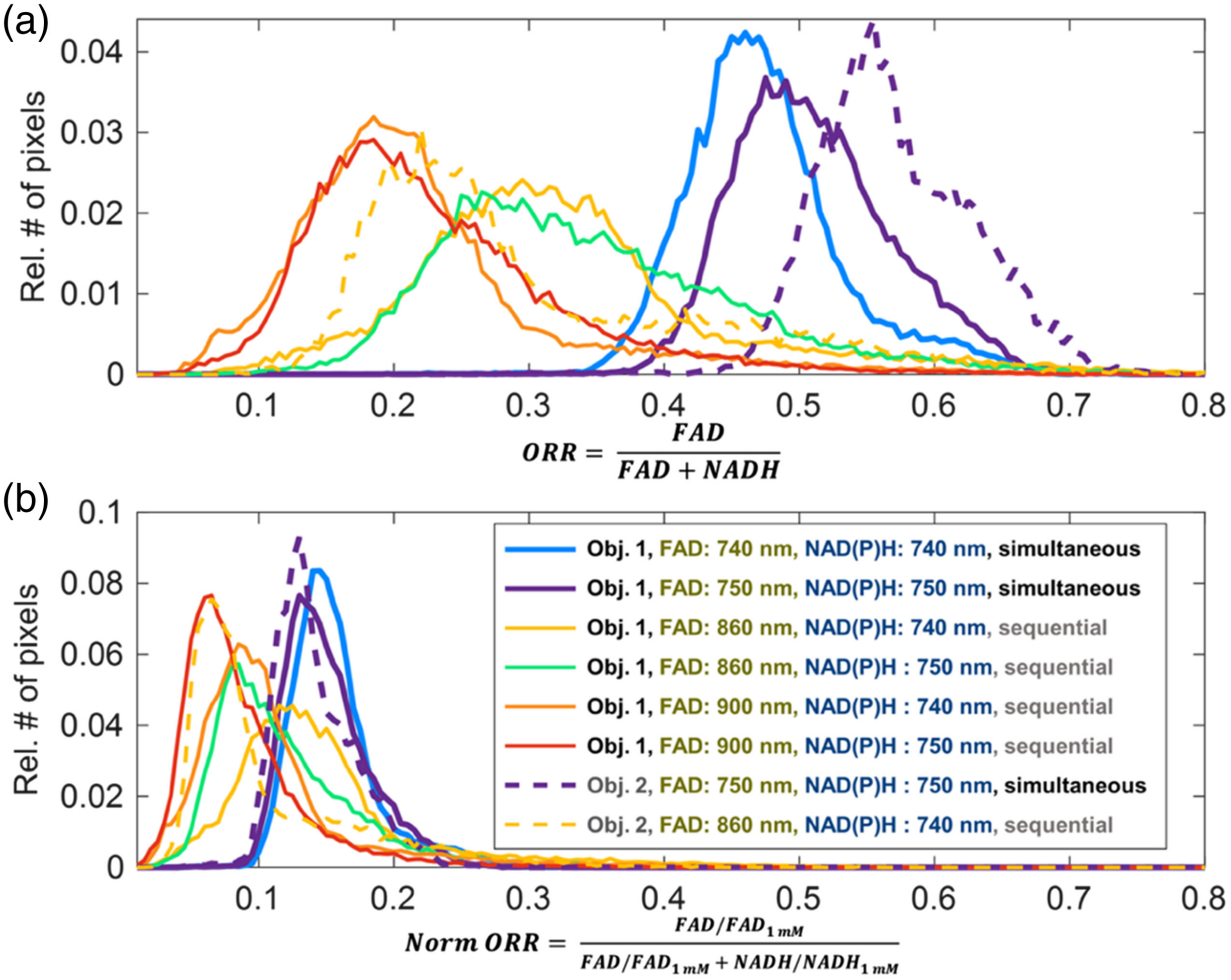
Optical redox ratio (ORR) values of cells with different acquisition parameters with and without normalization. ORR quantification of images shown in Fig. 4. (a) From FAD and NAD(P)H intensities (Fig. 4, row 5). (b) After normalization using 1 mM NADH and FAD standards (Fig. 4, row 6), ORR falls within a more consistent range and has more overlap between images acquired with different acquisition conditions. Images acquired using 740 nm or 750 nm excitation for FAD show higher ORRs due to the leakage of NAD(P)H signal into the FAD channel. However, there are still some differences between normalized ORRs across images.

To compare ORRs across imaging systems and/or research groups, calibrations of NAD(P)H and FAD autofluorescence intensity images may be performed with solutions of known NADH and FAD concentrations, as has been done previously.^64^^–^^69^ The relationship between fluorescence intensity and NAD(P)H or FAD concentration will vary due to factors such as NADH or FAD quantum yield changes due to protein binding, photon absorption, or scattering, as well as the presence of NADPH. Thus, the calibrated NAD(P)H and FAD fluorescence intensities cannot be associated with corresponding concentrations in many cases. However, calibration using standards with optical properties similar to the endogenous fluorophores facilitates comparisons of ORRs across time points and more importantly between different studies and microscopes. The importance of calibrating measurements with NADH and FAD solutions acquired under the same conditions as the sample images is demonstrated by computing redox ratios of a cell sample with images acquired using different excitation wavelengths and objectives in Figs. 4 and 5. As shown, the corresponding normalized ORR values vary over a significantly more confined range than the unnormalized ones. The remaining variations may be attributed, at least in part, to the fact that spectral overlap of the NAD(P)H and FAD excitation/emission was not taken into account when attributing all of the signal detected in the 535 to 605 nm region to FAD and all the signal detected in the 415 to 485 nm region to NAD(P)H for 740 and 750 nm excitation. This is particularly evident for the redox images computed by assuming that relevant NAD(P)H and FAD contributions can be distinguished based exclusively on emission wavelength separation, when a single excitation wavelength is used (blue and purple distributions-Fig. 5). Spectral unmixing approaches can be used for this purpose.^69^^,^^70^ The remaining variations likely originate from differences in the spectral excitation/emission properties of the free NADH and FAD solutions compared with the cellular fluorescence emanating from free and bound NADH and NADPH, and different flavins and flavoproteins. The presence of additional fluorophores in the cell (e.g., lipofuscin) can also yield some of the observed variations that are not accounted for fully by the calibration. Although these results demonstrate the importance of reporting the detailed calibration procedures and image acquisition parameters, we note that the level of accuracy achieved will depend on the sample.

As with imaging of any weakly autofluorescent species, achieving a strong signal-to-noise ratio (SNR) can be challenging for ORR imaging. Compared with FLIM analysis of NAD(P)H and FAD (see below), ORR measurements require less expensive instrumentation and are generally faster, simpler, and less sensitive to errors due to the overall poor SNR; but this depends on how data are summarized. Typically, investigators will ultimately compute average ORR from individual images, tissue regions, or cells. Averaging the NAD(P)H and FAD fluorescence intensities from a region of interest and then computing a redox ratio from those average fluorophore intensity values can improve the SNR of the ORR. This method of computing a redox ratio may have an advantage over computing an average of redox ratios from all relevant pixels, which can be skewed by pixels lacking the presence of NAD(P)H and FAD, if they are not removed from the calculations using thresholding to remove low-intensity pixels. On the other hand, pixel-wise redox ratio images may convey the spatial heterogeneity of metabolic function of the sample with high resolution, which is valuable for certain applications.

Variations in room temperature, pH, and atmospheric conditions (i.e., ${\mathrm{CO}}_{2}$ and $O_{2}$ levels) can affect significantly the metabolic state of the sample, especially when performing experiments using cell cultures, organoids, and engineered three-dimensional tissues. Thus, it is important to perform optical metabolic measurements when these environmental conditions are well-controlled, using, for example, stage top incubator systems. In addition, we note that standard, phenol-red-containing media used for cell culture have significant autofluorescence, which can interfere with optical metabolic assessments. Consequently, media free of components that yield endogenous fluorescence background with excitation/emission characteristics that overlap with those of NAD(P)H and FAD should be used for culture, if possible; alternatively, samples may be washed with such media prior to imaging and remain in that media during image acquisition.^45^ If possible, nuclear, lipid droplet, and intracellular or extracellular regions containing other fluorophores should be removed from ORR calculations, especially if such areas are significant relative to the size of the cell or tissue of interest. Depending on the cell or tissue type, investigators need to consider contributions from fluorophores such as lipofuscin, keratin, elastin, collagen, melanin, retinol, and carotenoids, which have overlapping excitation and emission fluorescence spectra.^6^^,^^71^ Spectral or multiwavelength imaging studies, as well as FLIM, can be used to elucidate and/or remove contributions from other fluorophores.^39^^,^^72^^,^^73^ If cellular regions containing additional fluorophores are included in the calculation, this will alter the redox ratio value. If the ORR is calculated from an average of pixel-wise redox ratios, then even areas lacking significant fluorescence (e.g., the nucleus) can potentially impact the average ORR due to changes in cellular or nuclear morphology (e.g., the cytoplasmic/nuclear ratio). More accurate ORRs from an average of pixel-wise redox ratios can often be achieved via segmentation of relevant cellular regions. Manual tracing and standard thresholding procedures (e.g., using Otsu’s method) are often performed for this purpose. Other segmentation techniques include automated identification of the cytoplasm using tools such as CellProfiler^74^ or deep-learning-based cellpose.^75^^–^^77^ If removal of noncytoplasmic regions is not possible because of SNR or resolution limitations, for example, this should be clearly reported and justified. Reporting redox ratio calculation and calibration procedures will improve transparency and provide important context for any SNR-related concerns or artifacts introduced by the presence of other fluorophores.

### 2.5 Essential Recommendations for Reporting Intensity-Based Redox Measurements

1. Perform measurements using consistent light sources, incident power, excitation and emission wavelengths, objectives, detectors, and detector gains and report such details in methods, along with image pixel size, and pixel dwell time and/or acquisition frame rate.
2. Normalize detected NAD(P)H and FAD signals by incident illumination power and any other variable instrumentation settings.
3. If a difference between ORR measurements is reported between a group of interest and corresponding controls as an outcome, control measurements should be acquired on the same day and also reported in the paper or its supplementary content.
4. Adopt a normalized form of the redox ratio to ensure the presence of a normal distribution of ORR values between 0 and 1 [Eq. (1) or Eq. (2)].
5. Include a sentence in the introduction to define the form of the ORR adopted and its relationship to changes in oxidative phosphorylation and glycolysis while also acknowledging that other metabolic pathways may affect this ratio, and there are alternative definitions.
6. Include the adopted ORR definition prominently in all relevant figures and/or its caption.
7. Acknowledge and consider potential impacts of other fluorophores and cellular features beyond the mitochondria and cytosol.

### 2.6 Best Practice Recommendations for Reporting Intensity-Based Redox Measurements

1. Normalize NAD(P)H and FAD-associated signals with corresponding images from NADH and FAD solutions for ORR assessments that facilitate comparisons among different studies and imaging systems.
2. Conduct segmentation to remove nuclear, lipid droplet, and lipofuscin-rich/lysosomal regions prior to reporting ORR. If that is not possible, clarify the reason why this was not necessary and/or acknowledge the potential limitations introduced by incorporating these regions in ORR calculations.
3. Perform measurements to ensure that most of the measured changes in autofluorescence are due to NAD(P)H and FAD under all relevant conditions (e.g., measure cofactor concentrations or include positive and negative controls using treatments that modulate metabolism).
4. Report SNR or SBR (signal to background ratio) values of NAD(P)H and FAD images.
5. Perform validation experiments using other techniques (e.g., respirometry, mass spectrometry, transcriptomic, proteomic, and metabolite measurements) to confirm origins of observed ORR changes.

## 3 Excited-State Fluorescence Lifetime-Based Measurements of Metabolic Function

### 3.1 Differences and Similarities Between Lifetime- and Intensity-Based Assessments of Metabolic Function

NAD(P)H and FAD remain the source of metabolic function contrast for FLIM imaging. FLIM is complementary to intensity-based measurements because it is sensitive to changes in chemical structures, fluorophore microenvironment, binding, aggregation, intermolecular interactions, and conformational changes. Some of the first measurements of NADH and FAD lifetimes in solutions were reported around the mid-20th century.^37^^,^^78^^,^^79^ This was followed by time-resolved spectroscopic lifetime measurements and association with metabolism—for example, distinguishing malignancy,^80^^–^^83^ or oxidation-reduction status in yeast.^84^ Advancements in fluorescence lifetime instrumentation ushered in the first autofluorescence metabolic FLIM studies using 1-photon excitation microscopy in yeast^85^ and two-photon excitation in Chinese hamster ovary cells.^86^ The first *in vivo* NAD(P)H FLIM study of metabolism in cancer progression was demonstrated in 2007.^60^ FLIM can be acquired by time-domain measurements such as time-correlated single-photon counting (TCSPC), time-gated cameras,^87^ and pulse sampling^88^^–^^90^ or frequency-domain techniques.^9^^,^^91^^,^^92^ Due to the widespread use of the TCSPC technique in FLIM studies, we will focus on this direct, versatile approach for analysis of the excited state dynamics within the context of the intrinsic fluorophores NADH, NADPH, and FAD.

FLIM can distinguish between the free and protein-bound states of NAD(P)H and FAD, providing insights into cellular metabolic states that can, in principle, be more sensitive to a range of subtle changes in the activity of different pathways to produce energy and synthesize critical biomolecules. For example, the NADH lifetime varies over ∼1 to 6 ns depending on the co-enzyme it is bound to,^6^^,^^9^^,^^93^ whereas differences between bound NADH and NADPH have also been reported.^94^^,^^95^ In most cases, the “bound FAD” signal is likely originating from a flavoprotein, whereas the “free FAD” signal is likely free FAD, but these interpretations are always context dependent.

FLIM is a self-referenced measurement that does not depend on fluorophore concentration (assuming sufficient photon counts), so it has some advantages over intensity-based imaging (e.g., no need for light throughput calibrations).^96^^,^^97^ Although metabolic function assessments using fluorescence intensity typically require measurements associated with both NAD(P)H and FAD, monitoring changes in FLIM signatures of one of the two fluorophores, typically NAD(P)H, is often sufficient to detect differences between two groups or conditions that can be reliably attributed to distinct metabolic states. However, the instrumentation to acquire FLIM data is more complex and expensive, and the acquisition time is longer than intensity measurements. In addition, analysis of FLIM images is more intricate than that of intensity images. Thus, experimental imaging conditions and analysis for performing FLIM of NAD(P)H and FAD with TCSPC should be carefully chosen, taking into account the experimental setup, the sample, and the biological question.

### 3.2 Key Considerations for FLIM Data Acquisition

Accurate FLIM measurements require recording of very fast processes that can be impacted by the response time of different instrumentation components, delays that can be introduced by cables in the system, and the readout speed of detected photons. The photon count rate for TCSPC experiments should be adjusted to avoid bias in lifetime estimates (pile-up effect) by ensuring a maximum acceptable photon rate of 5% to 10% of the excitation pulse rate, which is unlikely to be reached in NAD(P)H and FAD FLIM.^91^^,^^98^^–^^102^ As with intensity-based imaging, laser power should be adjusted to minimize phototoxicity in living cells and tissues, particularly for longitudinal imaging.^103^^,^^104^ Generally, the primary constraint for safe metabolic imaging is laser power as the photon rates required to avoid phototoxicity in living samples are usually low enough to avoid pile-up effects in the FLIM electronics. Recommended low laser powers (i.e., <5  mW in the sample plane for NAD(P)H) for safe metabolic imaging of live samples and low quantum yield of endogenous fluorophores generally result in 200,000 to 500,000 photons/sec and acquisition times of 10 to 100 s, depending on the number of pixels. The pixel dwell time and total integration time of the FLIM image should be adjusted to obtain the desired number of photons for accurate lifetime estimates.^105^ Finally, although for intensity-based measurements, there are different ways of combining the NAD(P)H and FAD images to assess redox, for FLIM, there are also distinct ways of analyzing the data to detect metabolic function changes.

### 3.3 FLIM Analysis: Multiexponential Decay Analysis

**Fig. 6:**
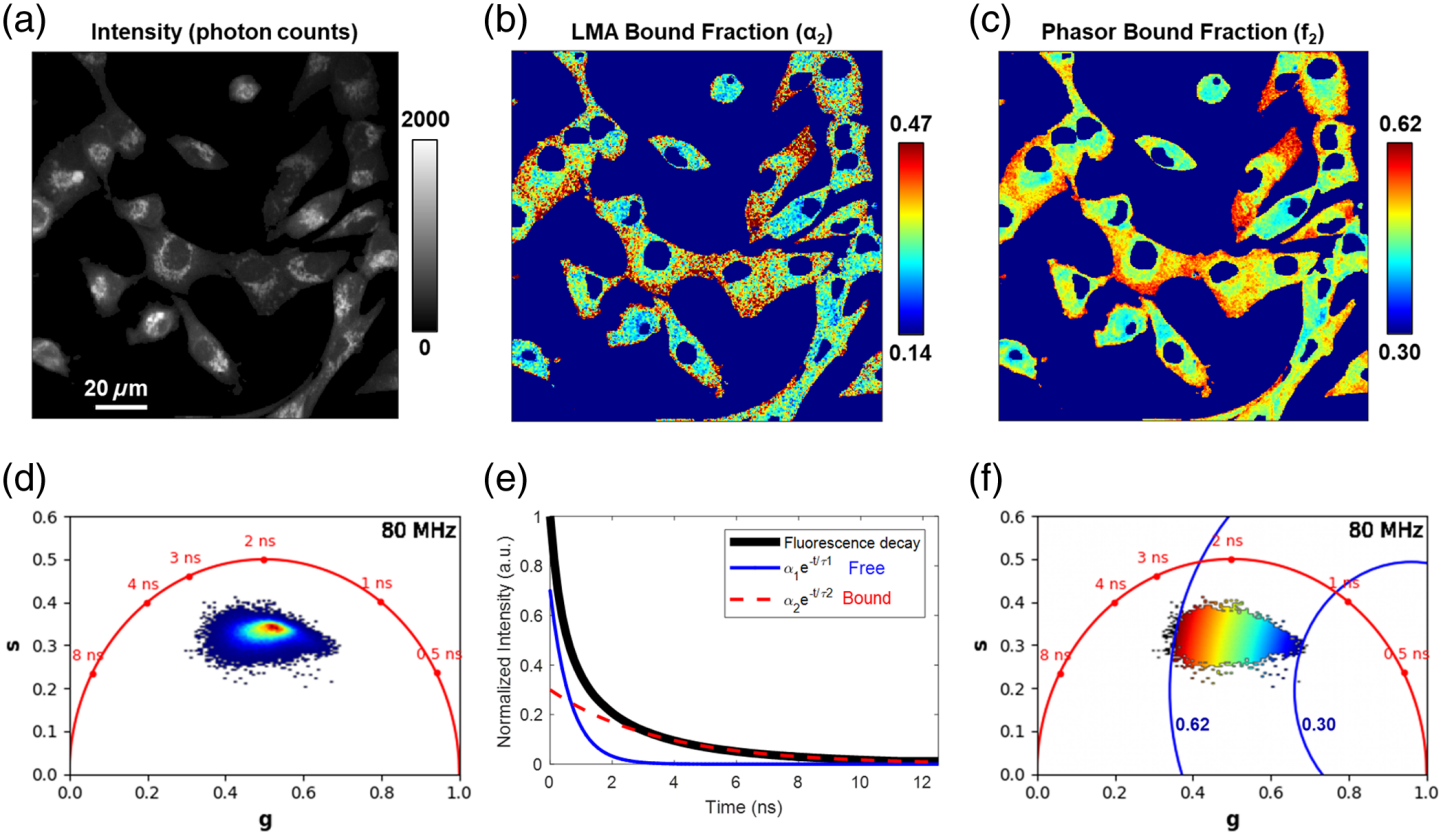
Biexponential fit and phasor analysis to examine the bound fraction of NAD(P)H *in vitro*. Biexponential fitting with Levenberg–Marquardt algorithm (LMA) using the open source FLIMJ software and phasor analysis via the open source FLUTE software were performed to analyze the two-photon FLIM of NAD(P)H in human breast cancer cells (MDA-MB-231) *in vitro*. (a) NAD(P)H intensity in photon counts. (b) Bound fraction of NAD(P)H estimated using FLIMJ. (b) Bound fraction of NAD(P)H estimated using FLUTE. (d) Unbiased intensity heatmap of phasor distribution created using FLUTE with no assumptions. (e) Free and bound components of the normalized mean fluorescence decay for all pixels in the image in the biexponential analysis performed with FLIMJ. (f) Phasor plot color-coded with distance from free NAD(P)H, assuming $\tau_{1}=0.4\;\mathrm{ns}$ and calculated by using the following formula $d=\sqrt{(g-g_{1}{)}^{2}+(s-s_{1}{)}^{2}}$ and created using FLUTE, with annotated distances in blue matching those of (c). NAD(P)H signal was collected centered at 451 nm using a bandpass filter (Semrock, FF01-451/106-25) with a photon-counting hybrid photodetector (PMA Hybrid-40, PicoQuant GmbH) on a custom two-photon imaging system (80 MHz, 25 mW, 750 nm excitation). Both open source software packages were calibrated with fluorescein in 100% ethanol (pH = 6.5 with 3.05 ns lifetime) and the IRF of the system. Each image size is 512×512  pixels with a field of view of 150×150  μm. Each image is the average of 20 frames, with a pixel dwell time of 8  μs for each frame acquired at a frame rate of 0.20 fps.

The type of FLIM analysis (Fig. 6) can be chosen by considering the number of photons collected in the FLIM image, desired processing speed and complexity of the biological sample and the fluorescence decay. The time-resolved fluorescence of NADH in a buffer exhibits a biexponential decay, which is attributed to different structural conformations based on the distance between the nicotinamide ring and the adenine base.^37^^,^^106^ Due to such structural flexibility, the fluorescence decay of NADH is sensitive to protein binding, which enhances the stretched conformation of this coenzyme and therefore yields a longer fluorescence lifetime (higher fluorescence quantum yield). The biexponential decay can be modeled as

$$I(t)=\alpha_{1}(e^{-t/\tau_{1}})+\alpha_{2}(e^{-t/\tau_{2}})+C,$$ (3)

where the fluorescence intensity at time t after excitation is represented as I(t). $\tau_{1}$ and $\tau_{2}$ are the fluorescence lifetimes of the short and long lifetime components, respectively, whereas $\alpha_{1}$ and $\alpha_{2}$ are the fractional contributions of the short and long lifetime components, respectively. C represents the background light contribution. The amplitude-weighted mean lifetime ($\tau_{m}$) from such a decay is defined as

$$\tau_{m}=\frac{a_{1}\tau_{1}+a_{2}\tau_{2}}{a_{1}+a_{2}}.$$ (4)

In living cells, populations of free and protein-bound NAD(P)H coexist at equilibrium, where the observed time-resolved fluorescence is often best described using two exponential decay components, fast for free NAD(P)H and slow for the enzyme-bound counterpart. The nature of the fluorescence decay is likely more complicated due to the heterogeneous local environments in living cells, different metabolic pathways being stimulated, site-specific refractive index, and the myriad of enzymes that rely on NAD(P)H for their biological functions. In addition, the slow fluorescence decay component depends on the type of enzyme bound to NAD(P)H.^6^ Biexponential decays have also been reported for cellular FAD; however, the bound FAD (likely a flavoprotein) exhibits a shorter fluorescence lifetime compared with the free counterpart.^6^^,^^85^

### 3.4 FLIM Analysis: Phasor-Based Analysis

Phasor analysis of FLIM is a fit-free method that converts the fluorescence decay of each pixel into a point on the 2D phasor plot. After the first formalization of the two-dimensional polar decomposition of lifetime measurements,^107^^,^^108^ the phasor plot was introduced^109^^,^^110^ and established as a powerful approach for FLIM by Digman et al.^111^ Phasor analysis can provide a graphical and unbiased representation of fluorescence decays, enabling easy and efficient visualization and interpretation of FLIM images,^9^^,^^112^^–^^116^ making it well-suited for complex and heterogenous samples such as tissue autofluorescence.^112^ However, quantification of specific changes in characteristic lifetimes and associated relative contributions remains challenging and still typically requires some additional knowledge or assumptions. For time-domain FLIM acquisition, the fluorescence decay, $I_{i,j}(t)$, at each pixel p(i,j) of the image can be transformed (via a Fourier cosine and sine transform) to corresponding coordinates $\left(g_{i,j},s_{i,j}\right)$ in the phasor plot using the following equations:

$$g_{i,j}(\omega )=\frac{\int_{0}^{T}I_{i,j}(t)\cos(\omega t)dt}{\int_{0}^{T}I_{i,j}(t)dt},$$ (5)

$$S_{i,j}(\omega )=\frac{\int_{0}^{T}I_{i,j}(t)\sin(\omega t)dt}{\int_{0}^{T}I_{i,j}(t)dt},$$ (6)

where f=1/T is the laser repetition rate and ω=2πf.

Phasor analysis can estimate the fluorescence lifetime based on phase ($\tau_{\varphi }=\frac{1}{\omega }\frac{s}{g}$) or modulation $\left(\tau_{\mathrm{mod}}=\frac{1}{\omega }\sqrt{\frac{1}{g^{2}+s^{2}}-1}\right)$, directly from g and s coordinates with no assumption on the lifetime components or species types. However, phasor analysis can also be employed to identify molecular species, their interaction, and their relative abundance using their decay similarity rather than determining fluorescence lifetime or decay components. Pixels exhibiting similar fluorescence decay characteristics cluster together in specific regions of the phasor plot. By analyzing the position and density of points in the phasor plot through manual^111^^,^^112^ or automated^117^^,^^118^ clustering, molecular species can be separated and identified within the fluorescence images. The linearity of the phasor space provides a graphical framework for quantifying the relative contribution of molecular species such as free and bound NAD(P)H.^111^^,^^112^^,^^119^^,^^120^ The phasor coordinates of the biexponential NAD(P)H decay (g,s) [Eqs. (5) and (6)] can be expressed as $g=f_{1}g_{1}+f_{2}g_{2}$ and $s=f_{1}s_{1}+f_{2}s_{2}$, where $\left(g_{1},s_{1}\right)$ and $\left(g_{2},s_{2}\right)$ are the phasor positions of free and bound NAD(P)H, respectively, and $f_{1}$ and $f_{2}$ represent their relative intensity contributions. The phasor positions of the free and bound species may be determined in an unbiased manner by fitting the phasor cloud of the image^43^^,^^92^^,^^114^^,^^121^^–^^123^ or alternatively, assigned to fixed values based on *a priori* assumptions regarding the pure species.^8^^,^^9^^,^^106^^,^^113^^,^^124^^–^^128^ Notably, the fit-free nature of phasor-based FLIM analysis provides a substantial advantage in computational efficiency compared with conventional multiexponential decay fitting.^114^^,^^116^^,^^129^^–^^132^ However, depending on the heterogeneity and complexity of the sample, the number of distinct decays, associated lifetimes, and fractional contributions that result in the calculated phasor cloud may still be difficult to identify accurately, and assumptions may be necessary to extract quantitative results that can be associated with specific metabolic pathway changes.

### 3.5 Calibration and Analysis Considerations

#### 3.5.1 Multiexponential decay analysis

The measured decay function, $I_{m}(t)$, is the convolution of the real fluorescence decay, I(t), with the Instrument Response Function of the measurement system, IRF(t):

$$I_{m}(t)=\int_{\tau =0}^{t}I(\tau )\mathrm{IRF}(t-\tau )d\tau .$$ (7)

The analysis procedure convolves the selected model function [e.g., Eq. (3)] with the IRF, compares the result with the measured decay data, and varies the amplitudes, $\alpha_{1}$, $\alpha_{2}$, and the lifetimes, $\tau_{1}$, $\tau_{2}$, until the best fit is obtained. This procedure is repeated for all pixels of the image. Decays are typically fit with the Levenberg–Marquardt (Weighted Least Square-WLS) or the maximum likelihood estimation (MLE) method, with MLE yielding more accurate lifetime assessments for fits of spectra associated with low photon counts.^102^

It is recommended that the fit parameters ($\tau_{1}$, $\tau_{2}$, $\alpha_{1}$, $\alpha_{2}$) and the corresponding distribution of fit parameters be clearly reported along with the SNR and binning procedure. SNR in FLIM can generally be represented by a Poisson distribution (i.e., ${\mathrm{SNR}}_{\mathrm{FLIM}}\propto \surd N$)^9^ or the F-value (normalized relative root mean square noise),^133^^,^^134^ so the number of photons per pixel should be reported at a minimum. It is also important to report the number of pixels per image, number of time bins per pixel, laser power at the sample, pixel dwell time, integration time, and the gain of the detector for reliable comparison with other studies. Thousands or more photons per decay are required, depending on the number of fit parameters, impulse/instrument response function (IRF), and experimental conditions,^102^^,^^105^^,^^135^ so pixel binning can be used to meet these minimum photon count thresholds but at the expense of spatial resolution. The threshold of the minimum photon count included in the fit analysis should also be reported. The fitting model (e.g., weighted least square, maximum likelihood estimation) should also be described. As the fluorescence lifetime of NAD(P)H depends on the type of enzymes involved as well as microenvironmental factors, it is not recommended to fix either fast or slow decay components observed in live cells or tissues based on data collected from buffered solutions of the fluorophores. NAD(P)H lifetimes may be sensitive to the exact location in living cells (nucleus, cytosol, or mitochondria) and presence of other fluorophores, so it is also helpful to show the $\chi^{2}$ distribution for a given FLIM image as an indication of the quality of the fitting model. In addition, the method for measuring the IRF and IRF full width at half maximum (FWHM) should be reported. Ideally, the IRF should be recorded under the same experimental conditions of laser wavelength and detector gain. The IRF is often measured with a second harmonic generation signal from a sample such as a urea crystal for two-photon microscopy^132^ or a fluorophore with a short lifetime like 2-DASPI with 30ps average lifetime (single photon microscopy).^136^ The IRF should be included with any publicly available datasets to enable proper reproduction of results. To ensure accurate decay fits, fluorescence standards should be used (e.g., coumarin, fluorescein, fluorescent beads), and recovered lifetimes should be comparable to published values.^9^ Several open source software tools are available for multiexponential decay analysis of FLIM images, such as FLIMfit,^137^ FLIM–FRET analyzer,^138^ Flimview,^139^ and FLIMJ.^129^ Details of the fit algorithm, including convolution, accounting for the impulse response function (IRF), and other considerations, are described in the TCSPC Handbook.^102^

#### 3.5.2 Phasor analysis

Similarly, for phasor analysis, a calibration standard such as a dye or the IRF is required to set the phase and amplitude shift of the phasors precision scales with SNR.^114^ Phasor analysis typically requires a few hundred photons per decay,^140^ and applying a median (or more complicated wavelet) filter to the phasor coordinates with a low photon budget effectively enhances the SNR without significantly compromising the spatial resolution.^119^^,^^141^^,^^142^ The frequency at which the Fourier transform is performed (i.e., first harmonic or higher harmonics) should also be provided. When phasor analysis is used to quantify the relative abundance of molecular species of interest, the lifetime or phasor locations of the pure molecular species (free and bound NAD(P)H) that are used to calculate the relative fractions should also be reported. The lifetime and phasor position of free NAD(P)H and FAD in solution are considered stable and are often used in Refs. 8, 9, 106, 113, and 124. The intensity fraction of bound NAD(P)H can be estimated by a two-component analysis when the location of bound NAD(P)H is assigned or extracted from a linear fit of the phasor cloud^43^^,^^92^^,^^114^^,^^121^^–^^123^ or by simply calculating the distance from the location of free NAD(P)H, typically assumed to be 0.4 ns.^125^^–^^128^ However, as noted above, the precise fluorescence lifetime can depend on a number of variable microenvironmental factors in addition to whether NAD(P)H is free or bound. Therefore, the method to estimate relative abundance should be clearly described.

To demonstrate similarities and differences between fit and phasor analysis of FLIM images, both approaches were applied to the same image (Fig. 6). Here, two-photon excited NAD(P)H [Fig. 6(a)] fluorescence lifetime images from human breast cancer cells are analyzed using bi-exponential fitting with a Levenberg–Marguardt algorithm (LMA) using the open source FLIMJ software^129^ [Figs. 6(b) and 6(e)] or using phasor analysis via the open source FLUTE software^130^ [Figs. 6(c), 6(d), and 6(f)]. Both open source software packages were calibrated with fluorescein in 100% ethanol (pH = 6.5 with 3.05 ns lifetime) and the IRF of the system. A mask was applied to the image to remove the background pixels with no cells, with a minimum photon count threshold of 200 photon counts. To increase the number of photons used for pixelwise lifetime calculations, raw images were summed with a 3×3 spatial filter prior to lifetime calculation, ensuring that 1800 or more photons were used for lifetime estimation in each pixel. The mean and median pixel-wise photon counts after binning were 3821 and 3048 photon counts, respectively. No temporal binning and no filtering were performed after lifetime analysis, though median filtering is commonly performed on fit lifetimes and/or phasor components. Nuclear regions were manually segmented. Fit parameters $\tau_{1}$ and $\tau_{2}$ and relative contributions $\alpha_{1}$ and $\alpha_{2}$ of free and bound NAD(P)H, respectively, were estimated for every binned pixel of the image, with mean values: $\tau_{1}=0.54\;\mathrm{ns}$, $\tau_{2}=2.81\;\mathrm{ns}$, $\alpha_{1}=2640$, $\alpha_{2}=1180$ [Figs. 6(b) and 6(e)]. For phasor analysis, the relative contribution of bound NAD(P)H $f_{2}$ was estimated by graphically calculating the distance of the experimental point from the phasor location of free NAD(P)H ($g_{1}=\frac{1}{1+(\omega \tau_{1}{)}^{2}}$, $s_{1}=\frac{\omega \tau_{1}}{1+(\omega \tau_{1}{)}^{2}}$), assumed to have a monoexponential lifetime $\tau_{1}=0.4\;\mathrm{ns}$, using equation $d=\sqrt{(g-g_{1}{)}^{2}+(s-s_{1}{)}^{2}}$ [Fig. 6(f)], whereas no assumption was made on the location of bound NAD(P)H. We note that the normalized fraction of bound NAD(P)H would be calculated with the following formula $f_{2}=\frac{\sqrt{(g-g_{1}{)}^{2}+(s-s_{1}{)}^{2}}}{\sqrt{(g_{2}-g_{1}{)}^{2}+(s_{2}-s_{1}{)}^{2}}}$, taking into account also the assigned position of bound NAD(P)H. The resultant contributions of bound NAD(P)H for both biexponential fit [Fig. 6(b)] and phasor [Fig. 6(c)] methods were within similar ranges and appropriate for NAD(P)H in cells. However, as noted above, when estimating the contribution of bound NAD(P)H, the biexponential fit provides the fractional contribution $\alpha_{2}$, whereas phasor analysis calculates the relative fluorescence intensity contribution $f_{2}=\frac{\alpha_{2}\tau_{2}\;}{\alpha_{1}\tau_{1}+\alpha_{2}\tau_{2}}$. As these two metrics are different, they cannot be directly compared, even though they yield similar information regarding metabolic function heterogeneity. As noted above, a biexponential decay of the lifetime or a specific short or long NAD(P)H or FAD lifetime is in most cases assumption that is not fully consistent with the complex fluorescence lifetime characteristics of cells and tissues; their impact on the reported results and associated interpretations should be thoughtfully considered. Several freely distributed and open source software tools are available to perform phasor analysis of FLIM data, such as SimFCS,^114^^,^^143^ PAM,^131^ FLIMJ,^129^ FLUTe,^130^ PhasorPy,^144^ GSLab,^145^ and FLIMPA.^146^

### 3.6 Metabolic Interpretations of Multiexponential and Phasor FLIM Analyses

Metabolic perturbations and comparisons to standard metabolic assessment techniques have been performed for both multiexponential decay and phasor analysis of NAD(P)H and FAD fluorescence lifetimes and have recently been reviewed.^6^ Anticipated changes in metrics such as the NAD(P)H bound fraction and mean lifetime are highlighted in Table 1 along with the ORR changes. For example, OXPHOS inhibition is routinely induced using rotenone or cyanide, which leads to an increase of free NADH,^43^^,^^126^^,^^128^^,^^147^^–^^150^ whereas mitochondrial uncoupling using Carbonyl cyanide p-(trifluoromethoxy) phenylhydrazone (FCCP)^43^^,^^148^^,^^149^ and glycolytic inhibition using 2-deoxyglucose (2DG) or glucose starvation^43^^,^^104^^,^^148^^,^^150^ lead instead to an increase of bound NADH. In addition, Pate et al. showed a correlation between the NAD(P)H phasor location and the ratio between oxygen consumption rate and extracellular acidification rate (OCR/ECAR) as well as ATP production in colon cancer cell lines.^151^ Cells with higher NAD(P)H lifetimes and higher proportions of bound NAD(P)H have a higher OCR/ECAR ratio, higher ATP production, and lower lactate concentration.^151^ Finally, Sanchez-Ramırez et al.^128^ observed a positive correlation between the proportion of bound NAD(P)H and basal respiration, maximal respiratory capacity, spare respiratory capacity, and ATP production in mesenchymal stem cells during adipogenic differentiation. Perturbations of FAD associated with metabolic pathways are less well characterized than NAD(P)H, but it has been shown that FAD lifetimes are sensitive to glycolysis inhibition.^150^

### 3.7 Essential Recommendations for Reporting FLIM-Based Metabolic Measurements

1. Perform measurements using consistent light sources, incident power, wavelengths, objectives, detectors, and detector gains and report such details in Methods, along with image pixel size, binning, and pixel dwell time or acquisition frame rate.
2. Ensure proper system calibration and report on details of IRF, fluorescence standard samples, and associated spectra; these measurements should be performed on the day of the experiment under similar laser excitation wavelengths and detector acquisition settings.
3. Employ strategies to achieve sufficient photon counts and report minimum and typical photon count numbers per decay analyzed.
4. Report analysis details in methods (thresholding, fit model used, binning, filtering approaches, frequency of phasor).
5. Report on the lifetime or phasor locations of the pure molecular species that are used to count the relative fractions.

### 3.8 Best Practice Recommendations for Reporting FLIM-Based Metabolic Measurements:

1. For time-domain analysis, report clearly the fit parameters $\left(\tau_{1},\tau_{2},\alpha_{1},\alpha_{2}\right)$ and the corresponding distributions along with the $\chi^{2}$ distributions for a given FLIM image as an indication of the quality of the fitting model.
2. Remove nuclear, lipid droplet, lipofuscin-rich/lysosomal regions prior to reporting FLIM-based analysis metrics that are supposed to be attributed to NAD(P)H and/or FAD. If that is not possible, clarify the reason why this may not be necessary and/or acknowledge the potential limitations introduced by incorporating these regions in your calculations.
3. Perform measurements to ensure that most of the signal is due to changes in NAD(P)H and FAD and all relevant conditions (e.g., measure cofactor concentrations or include positive and negative controls using treatments that modulate metabolism).
4. Perform validation experiments using other techniques (e.g., respirometry, mass spectrometry, transcriptomic, proteomic, and metabolite measurements) to confirm origins of observed FLIM changes.

## 4 Conclusion

This work contributes to the growing use of intensity and lifetime-based, label-free optical metabolic imaging of the intrinsic fluorophores NAD(P)H and FAD by discussing key considerations for reproducibility, comparison of data across groups, and metabolic interpretation. We provide clarity on the nomenclature regarding the ORR and its relevance to major metabolic pathways. In addition, we outline optimal practices for intensity and lifetime-based measurements, employing TCSPC acquisition and analysis methodologies such as multiexponential fit and phasor-based approaches. Our perspective underscores the importance of standardized methodologies in facilitating accurate metabolic interpretation and advancing the field of optical metabolic imaging.

## Data Availability

Data presented in Figs. 4–6 are available at https://figshare.com/projects/Optical_Metabolic_Imaging_Consensus_Paper/232223.
